# Multi-vendor robustness analysis of a commercial artificial intelligence system for breast cancer detection

**DOI:** 10.1117/1.JMI.10.5.051807

**Published:** 2023-04-18

**Authors:** Mercedes Riveira-Martin, Alejandro Rodríguez-Ruiz, Robert Martí, Margarita Chevalier

**Affiliations:** a Complutense University of Madrid, Medicine School, Department of Radiology, Rehabilitation and Physiotherapy, Madrid, Spain; b Galicia Sur Health Research Institute, Meixoeiro Hospital, Department of Medical Physics and RP, Vigo, Spain; c Nicolab BV, Amsterdam, The Netherlands; d University of Girona, Computer Vision and Robotics Institute (VICOROB), Girona, Spain

**Keywords:** mammography, breast cancer, screening, area under the curve, receiver operating characteristic curve, artificial intelligence

## Abstract

**Purpose:**

Population-based screening programs for the early detection of breast cancer have significantly reduced mortality in women, but they are resource intensive in terms of time, cost, and workload and still have limitations mainly due to the use of 2D imaging techniques, which may cause overlapping of tissues, and interobserver variability. Artificial intelligence (AI) systems may be a valuable tool to assist radiologist when reading and classifying mammograms based on the malignancy of the detected lesions. However, there are several factors that can influence the outcome of a mammogram and thus also the detection capability of an AI system. The aim of our work is to analyze the robustness of the diagnostic ability of an AI system designed for breast cancer detection.

**Approach:**

Mammograms from a population-based screening program were scored with the AI system. The sensitivity and specificity by means of the area under the receiver operating characteristic (ROC) curve were obtained as a function of the mammography unit manufacturer, demographic characteristics, and several factors that may affect the image quality (age, breast thickness and density, compression applied, beam quality, and delivered dose).

**Results:**

The area under the curve (AUC) from the scoring ROC curve was 0.92 (95% confidence interval = 0.89 − 0.95). It showed no dependence with any of the parameters considered, as the differences in the AUC for different interval values were not statistically significant.

**Conclusion:**

The results suggest that the AI system analyzed in our work has a robust diagnostic capability, and that its accuracy is independent of the studied parameters.

## 1 Introduction

Breast cancer is the most common cause of cancer death among women worldwide, as well as the cancer with the highest incidence.^1^ The implementation of population-based screening programs has significantly reduced mortality among women, as early detection of breast cancer increases the likelihood of successful treatment.^2^ However, these programs are based on 2D imaging diagnostic methods, which may lead to inaccuracies in terms of sensitivity (true positive rate) and specificity (true negative rate) as the overlapping of tissues can cause certain lesions to be hidden, while clusters of healthy tissue are perceived as lesions. To improve this aspect, screening programs recommend obtaining two views per breast: a craniocaudal (CC) and a mediolateral oblique (MLO) view, which increases the time taken by the radiologist to evaluate the examination. Screening programs are resource-intensive, both in terms of the time spent by radiologists reading mammograms and the financial cost.^3^ To facilitate the reading of mammograms, computer-aided diagnosis (CAD) systems were introduced in 1980. However, the detection capability of these systems relied on prior knowledge of the physical characteristics of breast lesions, which led to biased results, so the benefit of using CAD in screening is still unclear.^4^ On the contrary, with the introduction of artificial intelligence (AI), and mostly the introduction of deep learning (DL) algorithms, these systems have indeed proven to be an improvement, as several studies show that the diagnosis of the radiologists improves with the help of an AI system, with no need to lengthen the mammography reading time.^4^^–^^6^ Moreover, AI-based systems are unaffected by fatigue or subjective diagnosis.

Image quality can be defined on the basis of three important factors, including contrast, spatial resolution, and signal-to-noise ratio (SNR). These factors are in turn altered by parameters related to the image acquisition process, such as breast compression and thickness, which aims at minimizing scattered radiation and thus increasing contrast and SNR, positioning, the x-ray beam quality (determined by the anode/filter combination and voltage), and the radiation dose.^7^^,^^8^ Furthermore, these parameters may affect differently in the detection of different findings suggesting breast cancer, which are mainly soft-tissue lesions and calcifications.^9^^,^^10^ In the case of the former, the margins delimiting the lesion and its density are determinants of its malignancy, whereas in the case of calcifications, their number, morphology and distribution predominate.^8^ Thus, factors, such as contrast, may affect more severely the detection of soft-tissue lesions, whereas spatial resolution or noise may be more decisive in detecting calcifications.^11^^,^^12^

In addition, breast density (i.e., the percentage or absolute amount of fibroglandular tissue) is also known for being an essential factor affecting image quality. There are several studies claiming that the sensitivity of mammography screening is severely affected in women with high breast density, as the presence of heterogeneous or extremely dense tissue patterns may mask suspicious lesions.^13^^–^^15^ Moreover, studies have shown that women with high breast density are at higher risk of developing breast cancer and with more aggressive tumor characteristics,^16^ which highlights the importance of this property. The age of the woman plays also an important role in breast density, as it decreases with age and with the start of the menopause, although it has been shown that it is not an accurate surrogate for breast density.^17^ The most frequently used model among radiologists and screening examinations to classify breasts according to their density is the breast imaging-reporting and data system (BI-RADS), which classifies breasts into four categories: fatty, medium dense, heterogeneously dense, and extremely dense breasts.

Neural networks are susceptible to image quality-related parameters, such as noise distortions, contrast, blurring, or resolution.^18^^,^^19^ Therefore, just as image quality can affect image interpretation and detection of breast lesions by expert radiologists,^7^^,^^20^^,^^21^ it might also be an influential factor in the performance of a commercial AI-based CAD system. Currently, there are studies analyzing the effect of breast density on these systems,^22^^,^^23^ but to the best of our knowledge, the relationship of their performance with other parameters has not yet been investigated. The exponential development of AI systems applied to diagnostic imaging leads to the need to develop methods to evaluate their performance and determine whether there are parameters that may affect it. Consequently, the aim of this study is to evaluate the performance of a commercial AI system on a large cohort of screening exams as a function of different technical and demographic parameters.

## 2 Materials and Methods

On this study, the performance of a stand-alone AI-based screening tool is analyzed. For this purpose, the AI predictions on a particular set of screening examinations (inference) are compared against the clinical diagnostic on the same set (ground truth).

### 2.1 AI System

In this work, we have evaluated the robustness of the AI system marketed under the name Transpara^®^ (version 1.6.0, ScreenPoint Medical, The Netherlands). The model is composed of two modules that use DL convolutional neural networks, image analysis algorithms, and feature classifiers to detect both calcifications^24^^,^^25^ and soft tissue lesions (nodules or masses, distortions in the architecture of the breast parenchyma, and asymmetries).^26^^–^^28^ The soft tissue and calcification findings (showed as contoured and diamond marked regions, respectively) are subsequently combined to determine the suspicious region findings, assigning each region a value between 1 and 100, which represents the level of suspicion that the lesion is malignant (100 being the highest level of suspicion). Finally, dedicated algorithms are used to combine the scores of all regions detected on the right/left breast CC/MLO images into the exam-based score (AI score), which ranges from 1 to 10 (10 representing the higher probability that cancer is present). This score represents the overall probability of cancer on mammography, and it is calibrated so that approximately the same number of exams classified as normal (which is 10% of the total number of normal exams) falls in each AI score level (1 to 10).

The analyzed system works on full-field digital mammograms and is supported by examinations performed by mammography units of different manufacturers. At the moment of this study, the algorithm had been trained on 8800 biopsy-diagnosed cancer-positive exams, 5000 benign exams diagnosed by biopsy or by 1 year of patient follow-up, and 183,000 noncancerous exams, verified by 1 year of follow-up in the case of clinical diagnostic exams, or by 2 years of follow-up in the case of screening programs. These examinations were originated from devices from four different vendors (Siemens, Hologic, General Electric, Philips) and from institutions across Europe, United States, and Asia.^29^ Since Transpara is a commercial product and is not an open-source code, it is neither possible to provide further details on the model architecture nor on the train-test split that was used to train and validate the model. However, it is worth noting that none of the images that were used in this study had been previously seen by the algorithm during its training, validation, or testing.

### 2.2 Characteristics of the Study

This study has been carried out based on the results obtained when applying the AI system to a collection of mammography exams belonging to a population-based screening program, which were acquired consecutively between January and November 2018. Examinations within this time period were retrospectively collected from the picture archiving and communication system (PACS) without applying any additional selection criteria. The screening exams were all acquired at a single European institution with devices from two different vendors: Mammomat Inspiration (Siemens Healthineers, Forchheim, Germany) and Selenia Dimensions (Hologic Inc, Bedford, Massachusetts, United States), roughly 70% acquired with the former and 30% with the latter. To simplify, from now on, we will refer to these mammography units as “Vendor 1” (Siemens) and “Vendor 2” (Hologic). The imaging protocol consisted of the acquisition of two views per breast (CC and MLO) and the double reading of the mammogram by two radiologists independently. In total, six radiologists were involved in the screening reading in this sample. No information is available on interval cancers (cases diagnosed between consecutive screening rounds) or on the follow-up for normal exams.

Each examination was accompanied by the following details: the age of the woman, the thickness of the breast, the compression applied during the examination, the glandular dose, the density of the breast, the mammography unit in which the examination was performed, with its corresponding anode/filter combination, and the clinical diagnostic (ground truth), dichotomized as 0, without cancer, or 1 with cancer, corroborated by biopsy as the gold standard. For each patient, breast density was estimated independently for each of the four views (CC and MLO for each breast) as the volumetric breast percent dense tissue volume (PDV), using a DL algorithm that works on processed digital mammograms, developed by Vanegas et al.^30^ However, the mean value of both breasts in CC view was taken as the PDV for each patient, since the value in MLO view may be affected by the presence of the pectoral muscle in the images. The population characteristics are shown in Table 1.

**Table 1:** Overall demographic and examination-related characteristics of the examinations included in the study [median, range and interquartile range (IQR)]. They are shown for all the examinations, and for those obtained with the unit from Vendor 1 and from Vendor 2.

Parameter		Total	Siemens	Hologic
Number of exams		17,777	12,125 (68.2%)	5,652 (31.8%)
Cancer prevalence^a^		114 (6.4/1000)	81 (6.7/1000)	33 (5.9/1000)
Age	Median	58	58	57
	Range	48 to 69	48 to 69	50 to 69
	IQR	54 to 63	54 to 63	53 to 63
Thickness (mm)	Median	59	59	59
	Range	13 to 117	13 to 117	16 to 106
	IQR	49 to 68	49 to 68	48 to 68
PDV (%)	Median	10.15	9.86	10.78
	Range	4.55 to 44.22	4.55 to 42.34	5.07 to 44.22
	IQR	8.11 to 13.55	7.83 to 13.23	8.75 to 14.20
Compression (N)	Median	117	115	121
	Range	16 to 275	16 to 220	25 to 275
	IQR	100 to 140	99 to 138	100 to 145
Glandular dose (mGy)	Median	1.2	1.1	1.7
	Range	0.04 to 5.2	0.04 to 4.1	0.5 to 5.2
	IQR	0.9 to 1.6	0.9 to 1.4	1.2 to 2.1

a Ratio of positive cancer examinations, corroborated by biopsy as the gold standard.

**Fig. 1:**
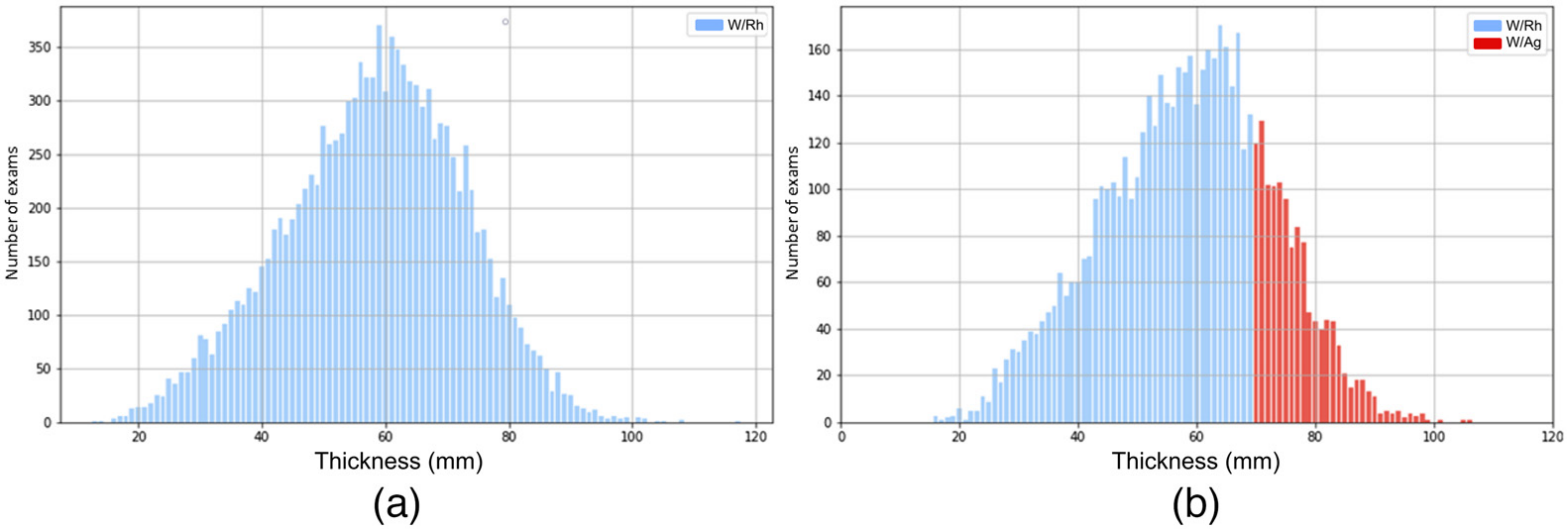
Distribution of exams performed with (a) Vendor 1 (Siemens) and (b) Vendor 2 (Hologic) and the anode/filter combination as a function of the breast thickness.

Regarding beam quality, Vendor 1 keeps the anode/filter combination of W/Rh for all examined breasts, regardless of thickness [Fig. 1(a)], whereas in the units from Vendor 2, the rhodium filter (W/Rh) is preferred for breasts thickness of less than ∼70  mm, and silver (W/Ag) for greater thicknesses [Fig. 1(b)]. The characteristics of both units (Vendor 1 and Vendor 2) are shown in Table 2.

**Table 2:** Technical characteristics of the different mammography units involved in the study.^31^^,^^32^

Vendor	Anode	Filter	Detector	Detector size	Detector pitch	Pixel array
Siemens	W	50 μm Rh	aSe	24.0×30.0 cm	85 μm	2800×3518
Hologic	W	50 μm Rh	aSe	23.3×28.7 cm	70 μm	3328×4096
		50 μm Ag				

a Abbreviations: aSe, amorphous selenium; W, tungsten; Rh, rhodium; Ag, silver.

### 2.3 Statistical Analysis

The sensitivity and specificity of the AI system has been studied by means of the area under the curve (AUC) of the receiver operating characteristic (ROC) curve. We have evaluated the influence of the following technical and demographic parameters on the results of the AI algorithm: the mammography unit manufacturer and the corresponding anode/filter combination (beam quality), the round of screening in which the woman is examined, the age of the patient, the compression applied, the thickness of the breast, the PDV, and the delivered glandular dose.

To this end, the AUC of the system was obtained for different intervals of these parameters and then the respective AUC values were compared to determine whether there are statistically significant differences between them. These intervals were defined as follows: two intervals separated by the median for the age, compression, thickness, and dose, and four for the density, separated on the basis of the quartiles. In all cases except for the density, the median was chosen as the threshold value to maximize the statistical power of the comparison, avoiding unbalanced intervals in number of exams (especially of cancer-positive tests). Particularly, breast density was divided based on the quartiles of the distribution to show the possibility of making a comparison of performance between four classes, which was inspired by the BI-RADS classification of breast densities. However, this does not imply a one-to-one relationship between the four intervals and BI-RADS category scales. In the case of the vendor, the anode/filter combinations and the round of screening, no intervals have been distinguished, but the comparison has been made between the AUCs obtained for each case (i.e., Vendor 1 versus Vendor 2; W/Rh versus W/Ag, etc.).

Spearman’s rank correlation coefficient (ρ) was used to obtain the correlations between the different parameters, to show that they are independent and therefore require separate analysis, assuming weak correlation for values 0≤ρ≤0.40, and strong correlation for values of ρ≥0.70.^33^ A Kruskal–Wallis test was performed to determine whether there were statistically significant differences between PDVs obtained from each view (CC and MLO). Bootstrapping was used to calculate the 95% confidence interval (CI) of the AUC. In the case of this study, bootstrapping analysis was also used to obtain the P-value (P) and to analyze whether there were statistically significant differences between two samples by hypothesis testing. For all statistical tests, we assumed a 95% confidence level, so significance was assumed if P<0.05. The libraries SciPy^34^ and scikit-learn^35^ from Python 3.8 have been used in the statistic analysis of this study and to obtain the AUC values of the ROC curves.

## 3 Results

### 3.1 Correlation between Parameters

The values of the Spearman’s rank correlation coefficients (Table 3) indicate a weak correlation between most parameters except for breast thickness and dose, especially in the case of Vendor 2. With the exception of these two parameters, the lack of correlation between the rest indicates that the analysis should be performed independently for each of them.

**Table 3:** Spearman’s rank correlation coefficient values (ρ) for the different parameter pairs for the total cohort of exams and for the exams performed by both vendors.

Parameters	Total	Siemens	Hologic
Age versus thickness	0.02	0.02	0.01
Age versus density	−0.23	−0.02	−0.23
Thickness versus compression	0.27	0.26	0.3
Thickness versus dose	0.66	0.66	0.85
Thickness versus density	−0.57	−0.59	−0.55
Compression versus dose	0.24	0.2	0.3
Compression versus density	−0.28	−0.27	−0.35
Dose versus density	−0.09	−0.09	−0.28

### 3.2 AI System’s Overall Performance

**Fig. 2:**
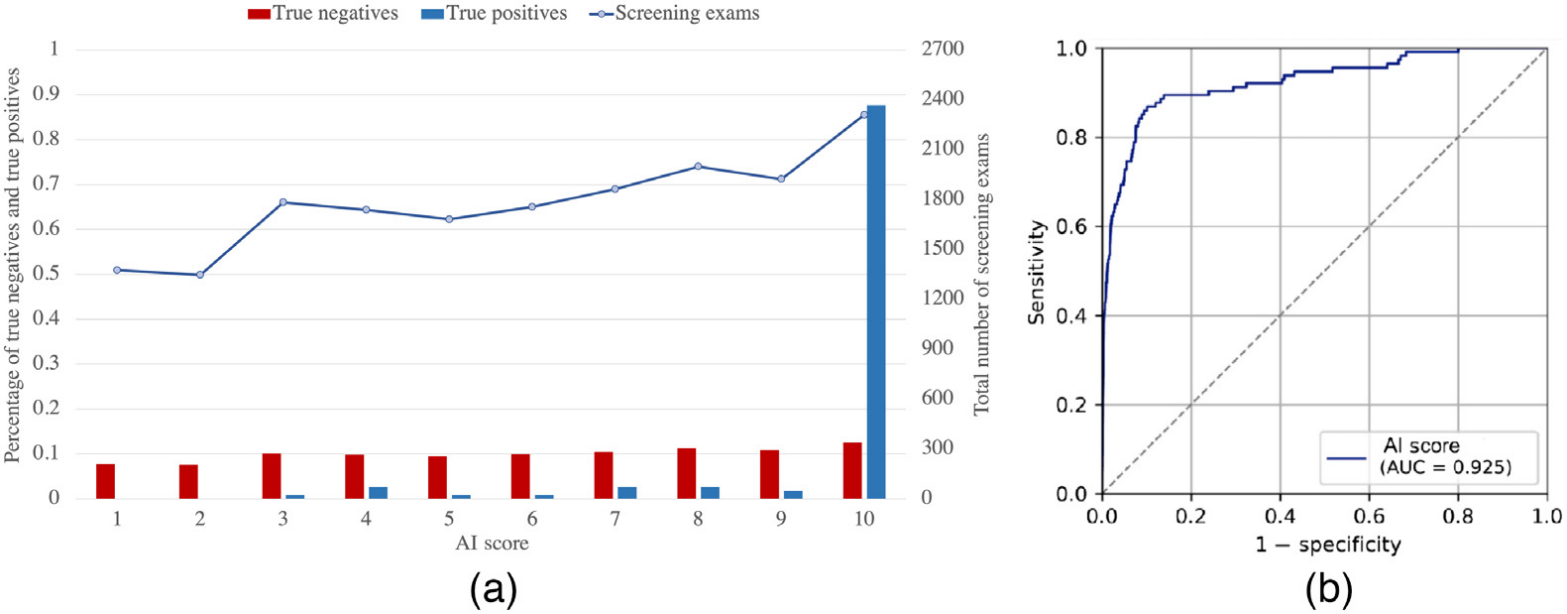
(a) Distribution of true negative, true positive, and total screen exams as a function of the AI system’s score (1 to 10). It is observed that normal tests are evenly distributed across all categories, with approximately 10% of all tests falling into each category, which is to be expected due to the calibration of the system. (b) ROC Curve and AUC value of the AI system evaluated over all exams.

The distribution of screening exams according to the AI score is shown in Fig. 2(b). As seen in the image, normal exams are evenly distributed throughout all the categories, with ∼10% of all the tests being grouped together in each one, which was expected due to the calibration of the system. The AI system classifies about 90% (87.7%) of all positive tests into category 10, which represents the maximum probability of the presence of cancer, and 96.5% into categories 5 to 10, both inclusive. The AI system’s overall AUC resulted in 0.92 (95% CI = 0.89 to 0.95). The ROC curve is represented in Fig. 2(a). For a threshold score of 10.0, the sensitivity and specificity values are obtained as 87.7% and 87.5%, respectively.

### 3.3 Dependence of the AI System with the Different Parameters

#### 3.3.1 Mammography unit manufacturer

**Fig. 3:**
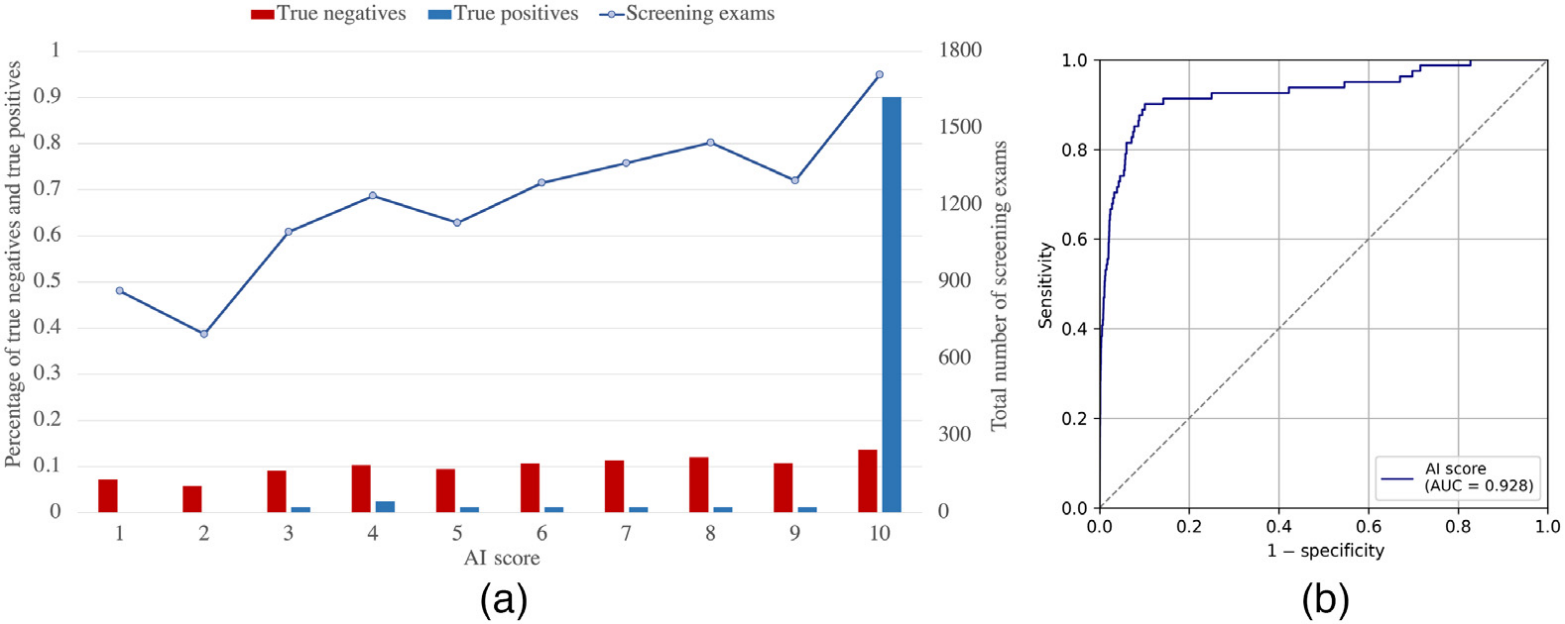
(a) Distribution of true negative, true positive, and total number of exams as a function of the AI score and (b) ROC curve and AUC value of the AI system for the examinations performed with Vendor 1 (Siemens).

**Fig. 4:**
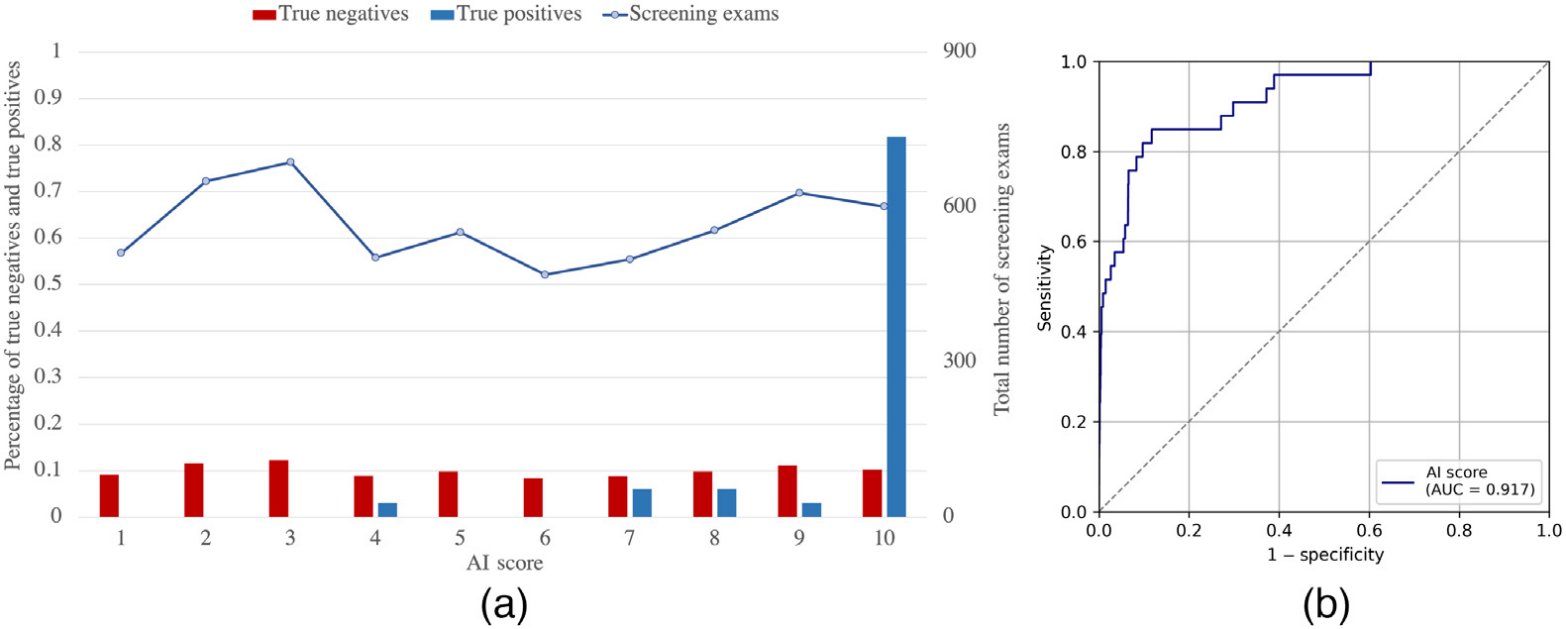
(a) Distribution of true negative, true positive, and total number of exams as a function of the AI score and (b) ROC curve and AUC value of the AI system for the examinations performed with Vendor 2 (Hologic).

The diagnostic capability of the system as a function of the mammography equipment (independent of image quality) was determined by the AI score of the examinations performed with each equipment. The median of the AI scores obtained with Vendor 1 (Me = 5.83) and Vendor 2 (Me = 4.86) was found to be significantly different according to the Kruskal–Wallis test (ΔMe=0.97, P<0.001). For a threshold value of 10.0, the sensitivity and specificity of the system with the exams performed by Vendor 1 is 90.1% and 86.4%, respectively, whereas for the examinations performed by Vendor 2 are 81.8% and 89.7%, respectively . However, as seen in Figs. 3 and 4, the distribution of screening exams as a function of the AI score is similar for both vendors. In addition, no significant differences (P>0.05) were found between the AUC of the AI system obtained only with the examinations performed with Vendor 1 (AUC = 0.93 (95% CI = 0.89 to 0.96)) from those with Vendor 2 (0.92 (95% CI = 0.86 to 0.96)), this difference resulting in 0.01 (95% CI=−0.05 to 0.07, P=0.48).

#### 3.3.2 Anode/filter combination

In the case of Vendor 2, the AUC of the model was obtained as a function of the anode/filter combination used (W/Ag or W/Rh, as shown in Table 2). The Kruskal–Wallis test indicates that there are not statistically significant differences between the scores of the two samples (P=0.85). In addition, the AUC with both combinations was obtained 0.90 (95% CI = 0.84 to 0.96) with W/Rh and 0.95 (95% CI = 0.93 to 0.98) with W/Ag, resulting in a not significant difference of 0.05 (95% CI=−0.005 to 0.104, P=0.47).

#### 3.3.3 First round of screening

**Fig. 5:**
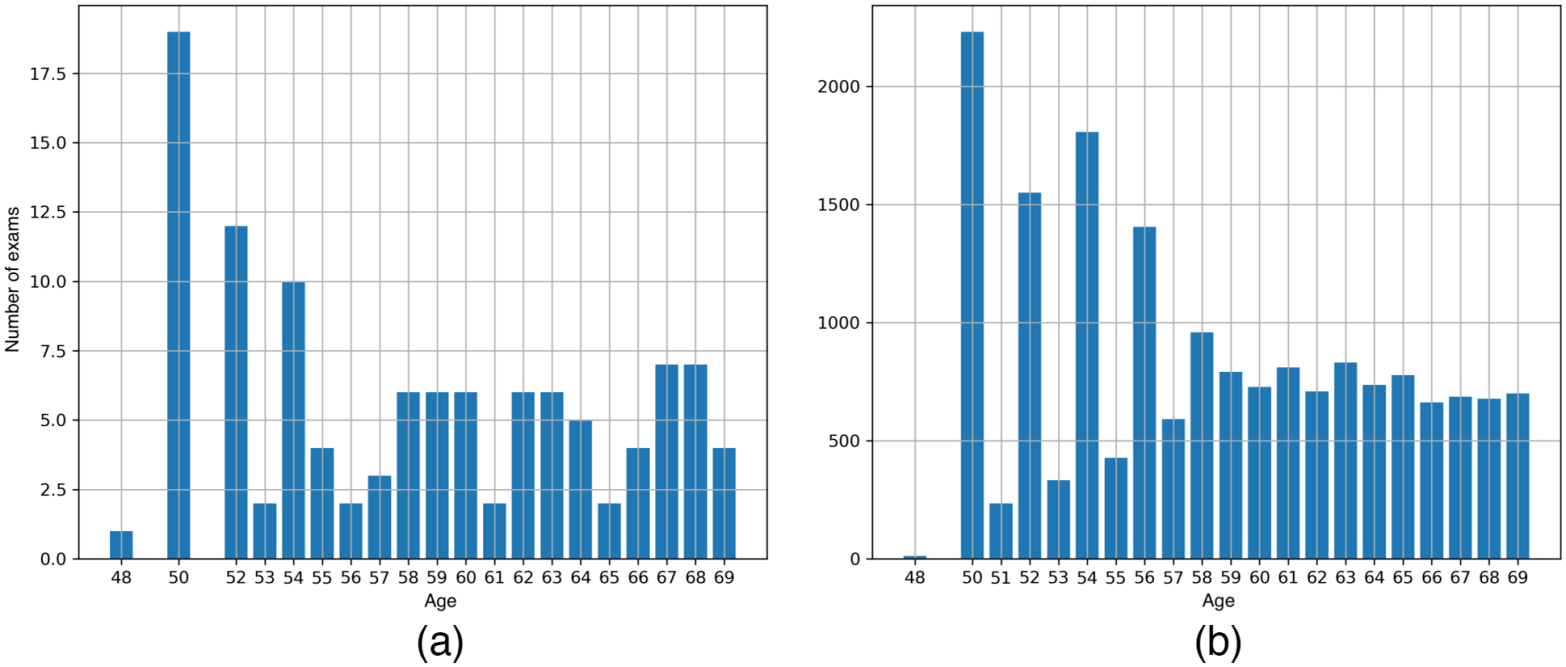
Histograms of the number of exams performed resulting in (a) cancer cases (truth = 1) and (b) noncancer cases (truth = 0) according to the age of the women considered in the study.

The distribution of screening examinations performed as a function of the age of the woman is shown in Fig. 5. It has been considered that women between 48 and 51 years old are initiated into the program, therefore being examined in their first round of screening. As seen in the figure, this round concentrates the majority of exams (both cancer and non-cancer cases). The AUC of the system was 0.92 (95% CI = 0.86 to 0.97) with the examinations from the first round and 0.92 (95% CI = 0.89 to 0.96) from the rest of the rounds, which resulted in an AUC difference of 0.0001 (95% CI=−0.060 to 0.072, P=0.48), considered not statistically significant.

#### 3.3.4 Age of the woman

**Fig. 6:**
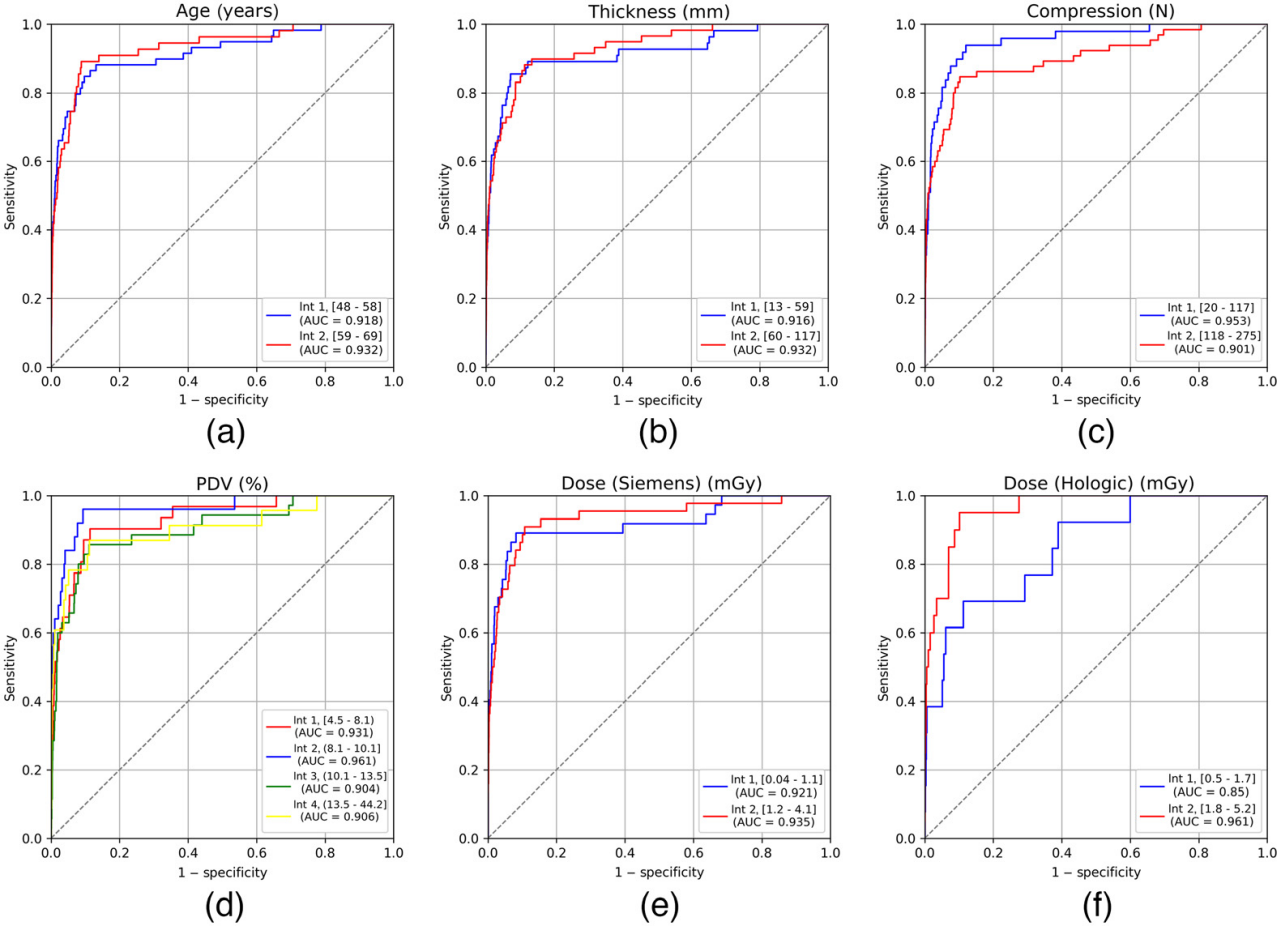
Comparison of the different ROC curves together with the corresponding AUC (legend) obtained for each of the intervals of each parameter, being (a) age of the woman, (b) thickness of the breast, (c) applied compression, (d) density in terms of PDV, (e) glandular dose from Vendor 1, and (f) glandular dose from Vendor 2.

The ROC analysis has been also performed by dividing mammography exams into two intervals according to the age of the woman [Fig. 6(a)]. The first group comprises women between 48 and 58 years old, as it has been previously shown that the first interval does not affect the performance, whereas the second group comprises women between 59 and 69 years old. The AUC of the system accounting for the exams from the first interval results in 0.92 (95% CI = 0.87 to 0.96) and for the second interval is 0.93 (95% CI = 0.89 to 0.97). The difference between both AUCs is not statistically significant, being 0.01 (95% CI=−0.05 to 0.08, P=0.51).

#### 3.3.5 Thickness of the breast

The AUC has been computed for two samples according to the thickness of the breast [Fig. 6(b)]. The first sample corresponds to low thickness breasts (from 13 to 59 mm) and the second to high thickness breasts (from 60 to 117 mm). It has been obtained that the AUC of the system for the examinations corresponding to the first interval (0.92, 95% CI = 0.86 to 0.96) does not show significant differences with the AUC from the second interval (0.93, 95% CI = 0.89 to 0.96), being this difference 0.01 (95% CI=−0.04 to 0.08, P=0.49).

#### 3.3.6 Compression applied

The values representing the compression applied to the breast during the examination were also divided into two intervals: low compression values (20 to 117 N) and higher values (118 to 275 N) [Fig. 6(c)]. The difference between the AUC of the AI system with data from the first interval (0.95, 95% CI = 0.92 to 0.98) and from the second interval (0.90, 95% CI = 0.85 to 0.94) was 0.05 (95% CI=−0.003 to 0.11, P=0.49), which is not statistically significant.

#### 3.3.7 Density of the breast

The AUC was obtained according to each of the four intervals into which the PDV was divided, and compared across all [Fig. 6(d)]. The AUC of the system for each interval and their comparison is shown in Table 4, where it can be seen that the difference between AUCs for each pair of intervals is not statistically significant.

**Table 4:** AUC values for each of the four density intervals and AUC differences (ΔAUC) between each pair of intervals. For visualization purposes, the comparisons between int 1 versus int 3 and int 4 versus int 3 are not shown, although they present AUC differences of 0.03 (95% CI=−0.05 to 0.11) and 0.001 (95% CI=−0.11 to 0.10), respectively, with P>0.05 for both. The P-value higher than 0.05 indicates that there are no statistically significant differences between intervals.

Interval	PDV range (%)	AUC	CI (95%)	Intervals	ΔAUC	CI (95%)	P
1	4.55 to 8.11	0.93	0.88 to 0.97	Int 2 versus Int 1	0.03	−0.03 to 0.07	0.50
2	8.11 to 10.15	0.96	0.91 to 0.99	Int 2 versus Int 4	0.05	−0.03 to 0.16	0.48
3	10.15 to 13.55	0.90	0.84 to 0.96	Int 2 versus Int 3	0.06	−0.02 to 0.14	0.49
4	13.55 to 44.22	0.91	0.81 to 0.98	Int 1 versus Int 4	0.02	−0.07 to 0.13	0.48

a Abbreviations: ΔAUC, difference between AUC for each interval.

#### 3.3.8 Delivered dose

We obtained the dependence of the AI system’s performance with the delivered dose according to the manufacturer of the unit. For each vendor, the dose values were divided into two intervals, as shown in Table 5. In the case of Vendor 1, the difference in AUCs from two intervals is 0.01 (95% CI=−0.06 to 0.09, P=0.485) [Fig. 6(e)] and in case of Vendor 2 this difference results in 0.11 (95% CI = 0.003 to 0.23, P=0.476) [Fig. 6(f)], both differences being not statistically significant.

**Table 5:** Dose intervals for both vendors. The range of the interval and the AUC of the system for the examinations within it are shown.

Vendor	Interval	Range (mGy)	AUC	CI (95%)
Siemens	1	0.04 to 1.1	0.92	0.85 to 0.97
	2	1.2 to 4.1	0.93	0.88 to 0.97
Hologic	1	0.5 to 1.7	0.85	0.73 to 0.95
	2	1.8 to 5.2	0.96	0.93 to 0.98

## 4 Discussion

Image quality has a major impact on the diagnostic rate of AI to evaluate breast cancer, as high-quality images may favor the detection and diagnosis of lesions.^36^ Therefore, in this work, we have evaluated the predictive ability of the AI system is independent of the mammography unit manufacturer (based on two vendors) Transpara and its potential dependency on certain parameters that may affect the image quality and thus, the prediction of the system.

Spearman’s rank correlation coefficients showed weak correlations between most of the analyzed parameters, except for breast thickness and dose, which show stronger positive correlation especially in the case of Vendor 2. This correlation was expected, as showed by other studies,^37^ as larger thicknesses require higher doses to maintain optimal image quality.^10^ In addition, thickness and density also showed an inverse weak correlation with the grade of breast density, which has also been showed in the literature.^37^ However, no pair of parameters showed such a strong correlation as to be excluded from the analysis.

The obtained AUC index of the system, accounting for all the examinations (0.92, 95% CI = 0.89 to 0.95) indicates high classification accuracy. In addition, the sensitivity (87.7%) and specificity (87.5%) values for a threshold score of 10.0, are in the range of the reference values of sensitivity and specificity for mammographic screening exams in the United States, which are 86.9% and 88.9% respectively, obtained from the Breast Cancer Surveillance Consortium (BCSC).^38^ The high proportion of correctly classified cases (category 10) indicates that the analyzed system is highly sensitive, which is consistent with the value of the AUC obtained from the ROC curve. This value is also consistent with other studies performed with Transpara for different sets of examinations.^4^^,^^6^^,^^22^^,^^29^^,^^39^

The values for sensitivity and specificity of the system for the examinations performed by each vendor are also similar to the reference values from the BCSC.^38^ Nevertheless, it is important to note that the number of examinations performed with Vendor 1 is considerably higher than with Vendor 2 (68% versus 32%, respectively). This implies that the data are unbalanced, which may explain why a statistically significant difference was found between the medians of the two groups and the lower AUC for Vendor 2 (Figs. 3 and 4). However, the ratio of positive cancer examinations is similar in both groups (~6/1000), which may explain that this difference was found to be not statistically significant. Therefore, we conclude that the diagnostic capability of Transpara, obtained in terms of sensitivity and specificity, does not depend on the mammography equipment used, in this case, Siemens and Hologic. In addition, it was shown that the anode/filter combination in the case of Vendor 2 units neither affects the accuracy of the system. These results are in agreement with other studies that use multicenter and multi-vendor data (Siemens Healthineers, Hologic, Philips, General Electric) to demonstrate the stand-alone breast cancer detection with Transpara.^4^^,^^29^

Breast cancer screening programs are performed at 2-year intervals. In the first round of screening, when women between 48 and 51 years old are initiated into the program, many of the lesions are seen for the first time, so radiologists refer more women to the specialist oncologist and more cancers are detected. That is why most of the examinations are concentrated on this first round, as seen in Fig. 5. Besides, it has been shown that breast cancer prevalence, the cancer detection rate, and all secondary screening mammography performance measures increase substantially with age.^40^ However, this study showed that neither the age of the woman nor the fact that the examination belongs to the first round of screening affects the accuracy of the system.

Compression, thickness, and glandular dose are also potential parameters that can affect image quality and thus the AI system capability, as they directly impact upon factors, such as image blurring or spatial resolution, which are crucial for lesion detection in the case of radiologists.^8^ However, we obtained that the AUC of the system was not affected by different values of these parameters. Nevertheless, regarding the glandular dose given with Vendor 2 [Fig. 6(f)], it is worth noting that the ROC curve for interval 2 (higher doses) comprises a larger AUC than for interval 1 (lower doses), in turn, making the difference greater (0.11 (95% CI = 0.003 to 0.23). This result may be due to the fact that the proportion of cases with cancer is lower in the low-dose interval (4.5/1000 versus 7.2/1000 in the first and second interval, respectively), so more cases would be needed to backup this result.

Breast density (PDV) is the only parameter that has been treated differently, dividing its distribution into four intervals. As indicated in previous sections, this classification was inspired by the BI-RADS classification. Initially, it was intended to establish a one-to-one relationship between interval boundaries and BI-RADS values reported by other authors, such as the Volpara density grades.^41^ However, since our data are not evenly distributed, the classes were not balanced in terms of the number of exams, thus diminishing the statistical power of the comparison. Nevertheless, it was found that the capacity of Transpara, in terms of AUC, does not vary according to the four defined density categories. This result is in agreement with another two studies that analyze the diagnostic capacity of Transpara as a function of density. In the study by Dustler et al.,^23^ they found no evidence of significant differences in the risk scores assigned to breast cancer cases in different BIRADS density categories, but point out that the system applies systematically higher scores for normal cases in certain BIRADS density categories. Lauritzen et al.^22^ found that the AI-based screening worked equally well across all breast densities.

Our results suggest that the AI system analyzed in this work (Transpara^®^, version 1.6.0, ScreenPoint Medical, The Netherlands) has a robust diagnostic capability, and that its accuracy is independent of the mammography equipment used, beam quality, screening round, woman’s age, breast thickness and applied compression, density defined as PDV, and glandular dose. As future work, it would be interesting to improve the statistical power of the study by increasing the proportion of cases with cancer, as well as to include information on the outcome of the system with benign lesions. With respect to breast density, it would also be worth performing the same analysis on the four BI-RADS densities, in addition to studying the dependence of other parameters that have not been considered in this study, such as the positioning, radiographic techniques, the race or ethnicity of the woman, the reproductive history, or history of cancer.

## 5 Conclusion

Population-based screening programs play a crucial role in the early detection of breast cancer, and AI-based systems represent a breakthrough in both speed and accuracy of diagnosis. This will enable more cost-effective scenarios in which the role of the human reader will change significantly. However, before being put into practice, it is necessary to thoroughly validate these systems and demonstrate that their diagnostic capability does not depend on factors related to either image quality or the screening population. The AI system analyzed in this work has been shown to be a robust and highly accurate model, whose diagnostic capability has not been affected by the parameters studied.

## Data, Materials, and Code Availability

The data that have been used to perform this study are not available, although the developed statistical models are available upon request.
